# Reversal of maternal obesity attenuates hypoxia and improves placental development in the preeclamptic-like BPH/5 mouse model

**DOI:** 10.32604/biocell.2023.029644

**Published:** 2023-09-28

**Authors:** Daniella M. ADAMS, Kalie F. BECKERS, Juliet P. FLANAGAN, Viviane C. L. GOMES, Chin-Chi LIU, Jenny L. SONES

**Affiliations:** 1 Veterinary Clinical Sciences, School of Veterinary Medicine, Louisiana State University, Baton Rouge, LA, USA; 2 Pennington Biomedical Research Center, Louisiana State University, Baton Rouge, LA, USA

**Keywords:** Preeclampsia, BPH/5, Leptin, Hypoxia

## Abstract

**Background:**

Women with obesity have higher risk of adverse pregnancy outcomes, including preeclampsia (PE). Late-gestational hypertension, aberrant fetoplacental development, and fetal growth restriction (FGR), hallmarks of PE, are observed spontaneously in BPH/5 mice. Similar to obese preeclamptic women, BPH/5 mice have higher visceral white adipose tissue (WAT) and circulating leptin. We hypothesized that attenuation of maternal obesity and serum leptin in pregnant BPH/5 mice will improve fetoplacental development by decreasing hypoxia markers and leptin expression at the maternal-fetal interface.

**Methods:**

To test this hypothesis, BPH/5 mice were fed *ad libitum* (lib) and pair-fed (PF) to C57 ad lib controls beginning at embryonic day (e) 0.5. Hypoxia-related genes, hypoxia inducible factor (Hif) 1α, stem cell factor (Scf), heme oxygenase-1 (Ho-1), leptin (Lep), and leptin receptor (LepR) were assessed in e7.5 implantation sites.

**Results:**

BPH/5 ad lib had 1.5 to 2-fold increase in *Hif1α*, *Scf*, and *Ho-1* mRNA and a greater than 3-fold increase in leptin mRNA *vs*. C57 that was attenuated with PF. Exogenous leptin promoted Hif1α and Ho-1 mRNA expression in e7.5 decidua *in vitro*. While hypoxic conditions *in vitro* did not change decidual leptin mRNA. Furthermore, BPH/5 PF mice demonstrated improved fetal and placental outcomes later in gestation, with greater placental vascular area by e18.5 and attenuation of FGR.

**Conclusion:**

In conclusion, pair-feeding BPH/5 mice beginning at conception may improve placental vasculature formation via decreased leptin and hypoxia-associated markers in this model. Future investigations are needed to better determine the effect of hypoxia and leptin on pregnancy outcomes in obese pregnant women.

## Introduction

Obesity is a widespread condition affecting one in three adults in the United States (Yang and Barouch, 2007). Pre-existing obesity in women increases the risk of developing gestational comorbidities, including diabetes mellites, hypertension, and preeclampsia (PE) (Catalano and deMouzon, 2015; Denison et al., 2010; Howell and Powell, 2017). PE is a pregnancy-specific syndrome that occurs in the second half of gestation clinically recognized by hypertension, proteinuria, and/or other signs/symptoms (American College of Obstetricians and Gynecologists, 2013). This can lead to adverse maternal and fetal outcomes. While the exact pathophysiology of PE is unknown, inadequate placentation with poor remodeling of spiral arteries is often seen in PE and results in decreased placental perfusion. This negatively impacts fetal development, as seen in mouse studies that have shown smaller litter sizes and fetal growth restriction (FGR) (Sones et al., 2016). This highlights the necessity of appropriate blood flow to prevent placental ischemia and allow for normal fetal growth (Gilbert et al., 2009). Vascular endothelial growth factor (VEGF) is a key factor during placentation that aids in angiogenesis at the fetal-maternal interface for appropriate oxygen and nutrient supply to the placenta. This has been shown to be lower in PE pregnancies along with higher circulating soluble VEGF receptor (R)1, sFlt-1, produced with pathologic placental hypoxia and scavenges free VEGF preventing placental angiogenesis (Nevo et al., 2006). VEGF overexpression within the endometrium of mice has been shown to parallel upregulation of the anti-angiogenic factor, sFlt1 (Fan et al., 2014). This has also been observed in rodent models of PE (Woods et al., 2011; Reijnders et al., 2018).

To study the basic mechanisms of PE, we utilize a mouse model, borderline blood pressure high (BPH)/5 (Davisson et al., 2002). Female BPH/5 mice are prehypertensive and obese due to hyperphagia, and spontaneously develop a preeclamptic-like phenotype in pregnancy (Davisson et al., 2002; Sutton et al., 2017). Additional studies revealed overexpression of decidual VEGF in *ad libitum* fed BPH/5 female mice implantation sites at e7.5 prior to higher circulating sFlt-1 and lower placental VEGF at mid-gestation compared to control mice (Olson et al., 2020; Reijnders et al., 2018; Woods et al., 2011). BPH/5 mice exhibit FGR with lower pup and placenta weight with placental oxidative stress, and decreased litter size during pregnancy compared to control C57 mice (Dokras et al., 2006; Reijnders et al., 2019; Davisson et al., 2002). When BPH/5 mice are pair-fed to lean normotensive control mice beginning at conception they have normalization of decidual VEGF (Olson et al., 2020).

During early gestation hypoxia is necessary to promote trophoblast cell invasion and the formation of spiral arteries to increase oxygen delivery. Hypoxia stimulates the induction of different pathways, including the transcription of hypoxia inducible factor (HIF) which includes a constitutively expressed ß subunit and an oxygen-dependent α subunit (Soares et al., 2017). Activation of HIF leads to the expression of genes that encode proteins which control adaptations to hypoxia. Other pathways that serve as markers of hypoxia include stem cell factor (SCF) and heme oxygenase-1 (Ho-1), which we investigated in this study. Levels of HIF α correspondingly stabilize during early pregnancy with the occurrence of placentation. While maternal hypoxia is physiologic at specific time points in gestation, severe, prolonged, or transiently sensitive exposure to maternal hypoxia can have detrimental consequences to the fetoplacental unit (Soares et al., 2017). Such effects include shallow trophoblast invasion, narrowing and higher resistance placental arteries, which can lead to conditions such FGR and PE (Soares et al., 2017). Finally, previous studies have demonstrated increased leptin in pregnant females with PE, and that leptin gene expression is elevated during hypoxic conditions (Grosfeld et al., 2001). Elevated circulating leptin in women with PE may serve as physiological signaling for more nutrients due to decreased placental perfusion (de Knegt et al., 2021). This is an area of important research in the field. This was tested *in vivo*, which demonstrated that hyperleptinemic BPH/5 pregnant mice that are pair-fed have lower leptin along with reduced visceral adipose tissue (Reijnders et al., 2019). Furthermore, maternal weight loss and lower circulating leptin has been shown to be associated with attenuation of late-gestational hypertension and FGR (Beckers et al., 2023). However, the precise mechanism is under investigation. We hypothesized that leptin signaling contributes to excess hypoxia in BPH/5 decidua in early gestation resulting in poor placental blood flow and downstream adverse maternal and fetal outcomes.

To test our hypothesis, we measured hypoxia-related factors in early gestation implantation sites from BPH/5 *ad libitum* and pair-fed pregnant mice and compared to controls. Placental leptin expression and vasculature was assessed by histology after pair-feeding BPH/5 was continued into late-gestation to complement attenuation of FGR as previously described in this model.

## Materials and Methods

### Animal experiments

Adult (8–12 weeks of age) female and male BPH/5 and C57 mice were used in this study. BPH/5 mice were a gift from Dr. Robin Davisson, Cornell University and are maintained as an in-house colony at Louisiana State University. Mice were housed in Micro-Isolator^™^ cages (Lab Products, LLC. Aberdeen, Maryland, USA) on individually ventilated cage racks (Lab Products, LLC. Aberdeen, Maryland, USA) placed in a temperature- and humidity-controlled facility, maintained on a 12-h light/dark cycle, and fed standard mouse chow (LabDiet 5001 Laboratory Rodent Diet, LabDiet, St. Louis, MO, USA) with water available *ad libitum*. Intra-strain timed matings were performed in C57 and BPH/5 with the day of vaginal copulatory plug detection designated as embryonic day (e) 0.5. *Ad libitum* fed C57 (n = 5) and BPH/5 mice (n = 5) had food intake monitored daily. Another cohort of plugged BPH/5 female mice (n = 8) were pair fed, such that they were fed matching chow quantities consumed by C57 gestation-matched female mice beginning at e0.5 (approximately 25% calorie reduction in early and mid gestation) until end points e7.5 and e18.5 as previously described (Reijnders et al., 2019; Sutton et al., 2017). Mice were then sacrificed and implantation sites or placenta collected for molecular and histologic analyses. All procedures were approved by Louisiana State University’s Institutional Animal Care and Use Committee. The mouse studies met the standards set forth by the National Institutes of Health guidelines on care and use of animals, United States Department of Agriculture regulations, and the American Veterinary Medical Association Panel on Euthanasia.

### Quantitative reverse transcription PCR (qRT-PCR)

Total RNA was extracted from snap frozen BPH/5 and C57 e7.5 implantation sites (n = 5–10 per pregnancy) using Trizol (Thermo Fisher, Waltham, MA) according to manufacturer guidelines. cDNA was synthesized and relative expression of hypoxia-related genes (Lep, Lepr, Scf, Ho-1, Hif1α) was determined by qRT-PCR using SYBR Green (Qiagen, Hilden, Germany) as previously described (Reijnders et al., 2018). All primer sequences have been used previously and are listed in Table 1. Each qRT-PCR was performed in triplicate with 25 ng cDNA. The relative expression levels (2^−ΔΔCt^) relative to 18S rRNA (Reijnders et al., 2018).

### Leptin ELISA

Placenta lysates were prepared in lysis buffer with proteinase inhibitor using a commercially available Leptin ELISA and performed according to manufacturer’s instructions (Cayman Chemicals, Ann Arbor, MI; catalog #10007609) as previously described (Reijnders et al., 2019). The sensitivity of the assay is 50 pg/mL.

#### Placenta histology:

At e18.5, BPH/5 *ad libitum* and pair-fed pregnant mice (n = 3/group) had placenta dissected and fixed in formalin prior to processing for histological analysis. Masson’s trichrome stain was performed as previously described (Dokras et al., 2006). Decidual vessels in transverse section (n = 5–10 per placenta) were used for area measurement using Zeiss zen blue software.

### Decidua explant culture

Implantation sites (n = 8) from e7.5 ad lib C57 mice and ad lib BPH/5 mice were excised and dissected free of the serosa and myometrium layers prior to *in vitro* analyses. Decidua was placed in DMEM culture media supplemented with 10% fetal bovine serum and antibiotics at 37°C. Leptin (100 nM; Peprotech, Rocky Hill, NJ, USA) was added to the culture media (n = 3). After 16 h, the tissue was snap frozen in cryotubes and stored at −80°C. Remaining e7.5 decidua from ad lib C57 and ad lib BPH/5 mice were placed in a vacuum bag, ambient air was evacuated, and the bags were sealed with a vacuum sealing machine as previously described (Matthiesen et al., 2021). These conditions have been shown to mimic hypoxic conditions *in vitro*. Tissues were snap frozen in cryotubes with liquid nitrogen then stored at −80°C after 5 h incubation. RNA was isolated from decidual tissue and qRT-PCR performed as described above.

### Statistical analysis

GraphPad Prism version 7 software was used to assess the statistical significance (*p* < 0.05) between groups with a *t*-test or a one-way ANOVA when appropriate. All data was illustrated as the mean of the samples +/− standard error of the mean (SEM).

## Results

### Hypoxia-related factors and leptin mRNA is upregulated in pregnant BPH/5 e7.5 implantation sites and is attenuated with pair feeding

**FIGURE 1.**
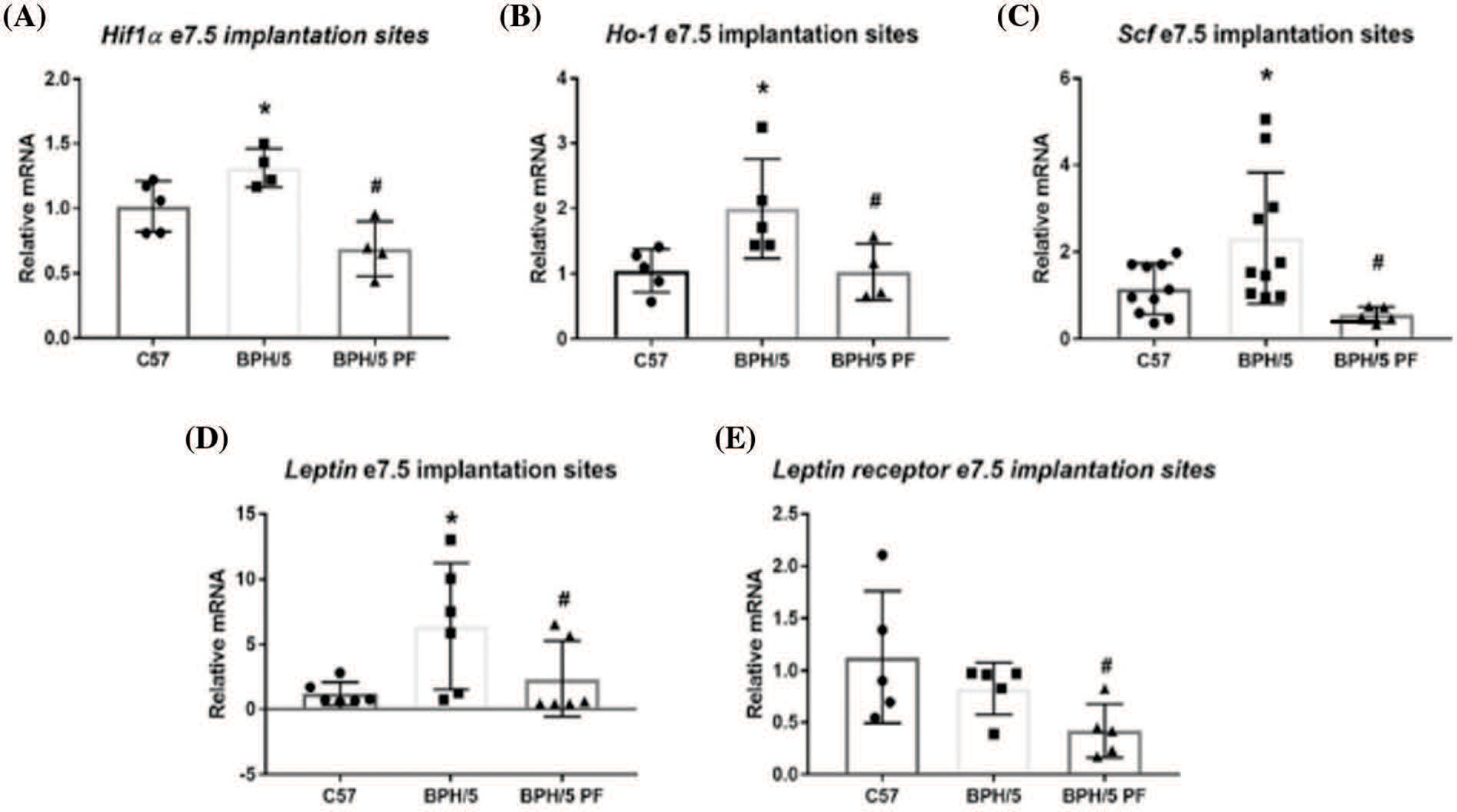
BPH/5 e7.5 implantation sites exhibit increased hypoxia markers including leptin in comparison to C57b, which normalizes with pair-feeding (PF). Qualification of hypoxia marker mRNA expression by qRT-PCR in ad libitum (lib) C57 and BPH/5, PF BPH/5 e7.5 IS (n = 5–10/group). Hypoxia markers evaluated included (A) *Hifla*, (B) *Ho-l*, and (C) *Scf*. Quantification of (D) leptin and (E) leptin receptor mRNA expression by qRT-PCR in C57, ad lib BPH/5, PF BPH/5 e7.5 implantation sites (n = 5–10). *p < 0.05 *vs*. C57 ad lib, #*p* < 0.05 *vs*. BPH/5 ad lib.

Previous studies have noted an angiogenic imbalance in BPH/5 placenta and implantation sites, which is normalized by pair-feeding (Olson et al., 2020). To investigate if hypoxia-related factors have a similar expression pattern, BPH/5 and C57 e7.5 implantation sites were assessed for the expression of hypoxia markers using qRT-PCR analysis. Ad lib BPH/5 mice demonstrated a 1.5 to 2-fold higher expression of *Hif-1α*, *Scf*, and *Ho-1* mRNA in comparison to C57 mice (*p* < 0.05) (Figs. 1A–1C). To further determine if maternal obesity was associated with elevated hypoxia, expression in pair-fed BPH/5 e7.5 implantation sites were assessed. Normalization of mRNA levels of all hypoxia markers, *Hif-1α*, *SCF*, and *Ho-1* was observed in comparison to *ad libitum* (lib) fed BPH/5 mice (*p* < 0.05) (Figs. 1A–1C). Comparable to hypoxia marker expression, ad lib BPH/5 demonstrated a greater than 3-fold higher *Lep* expression compared to C57 control mice which was reduced with pair-feeding (Fig. 1D). *LepR* expression was not significantly different in e7.5 ad lib BPH/5 implantation sites compared to control mice but did demonstrate decreased expression after pair-feeding BPH/5 (Fig. 1E).

### Decidual hypoxia gene expression in BPH/5 is sensitive to leptin in vitro

**FIGURE 2.**
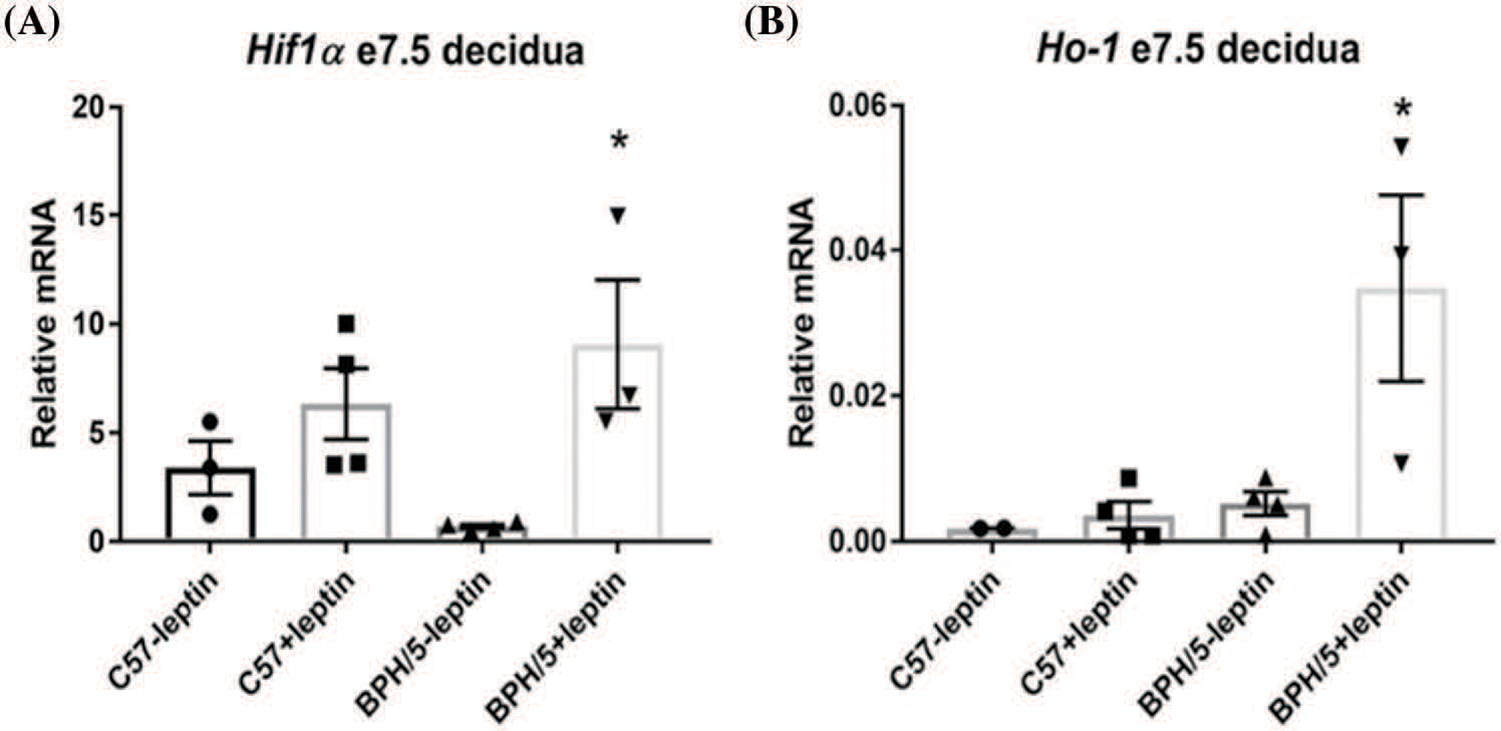
Leptin directly promotes hypoxia markers in the BPH/5 early gestation desidua. (A) Quantification of hypoxia marker mRNA expression by qRT-PCR in ad libitum (lib) C57 and BPH/5 e7.5 decidua *Hifl α* and (B) *Ho-l* (n = 3–4). *p < 0.05 *vs*. C57 ad lib and BPH/5 ad lib.

Previous studies demonstrated increased circulating leptin in pregnant BPH/5 mice compared to C57 controls (Reijnders et al., 2019). Because our previous results also demonstrated that BPH/5 mice have elevated *Hif-1α*, *Scf*, and *Ho-1* expression in e7.5 implantation sites, we investigated if elevated leptin influences the upregulation of these hypoxia markers in the maternal decidua specifically. We assessed *Hif-1α*, *Scf*, and *Ho-1* mRNA expression in e7.5 C57 and BPH/5 decidua when cultured with exogenous leptin. Interestingly, only BPH/5 e7.5 decidua showed a significant elevation of *Hif-1α* and *Ho-1* mRNA with >6-fold increase in expression when cultured with leptin compared to BPH/5 e7.5 decidua cultured without leptin (Figs. 2A and 2B). Incubating e7.5 decidual explants in hypoxic chambers failed to significantly change leptin gene expression in C57 (mean delta Ct = 17.1) nor BPH/5 (mean delta Ct = 15.1 *vs*. normoxia mean delta Ct = 15.85).

### Placental vessel area increases in BPH/5 pregnant dams with pair-feeding

**FIGURE 3.**
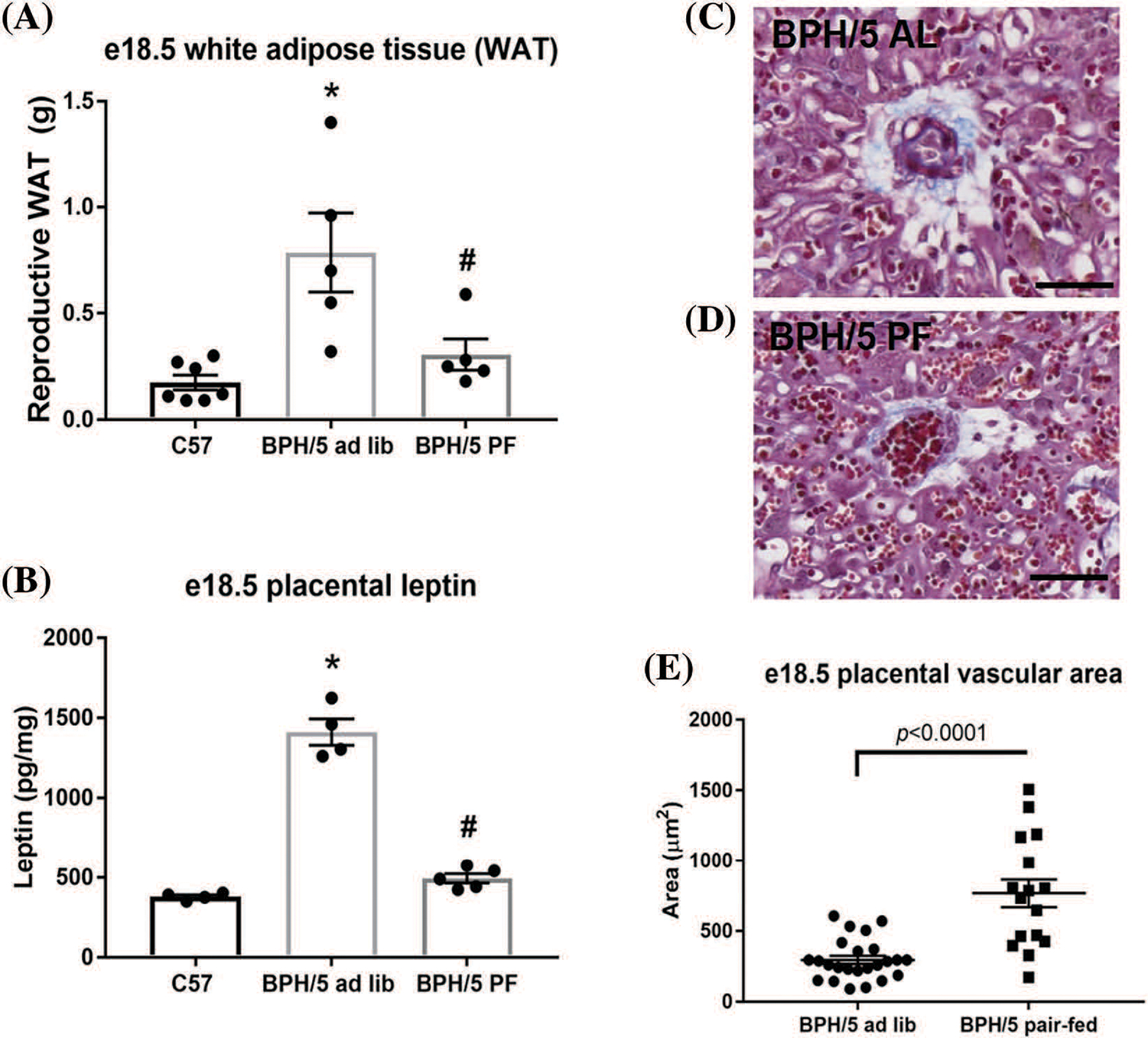
Decreased placental vasculature size at e18.5 in ad lib BPH/5 mice demonstrate improvement with pair-feeding (PF). (A) Measurements of white adipose tissue (WAT) around the reproductive tract (n = 4–8 dams) and (B) quantification of placental leptin protein expression by ELISA (n = 4–5) from ad libitum (lib) C57 and BPH/5, and PF BPH/5. *p < 0.05 *vs.* C57 ad lib; ^#^*p* < 0.05 *vs.* BPH/5 ad lib. Representative image of a transverse section of Masson’s trichrome stained e18.5 placenta from (C) ad lib (AL) and (D) pair-fed (PF) BPH/5 pregnancies. The blue color denotes the stained collagen, which signals the presence of vessels. (E) Quantification of placental vasculature area through Zeiss zen blue software (n = 3/group). Scale bar = 50 μm.

**FIGURE 4.**
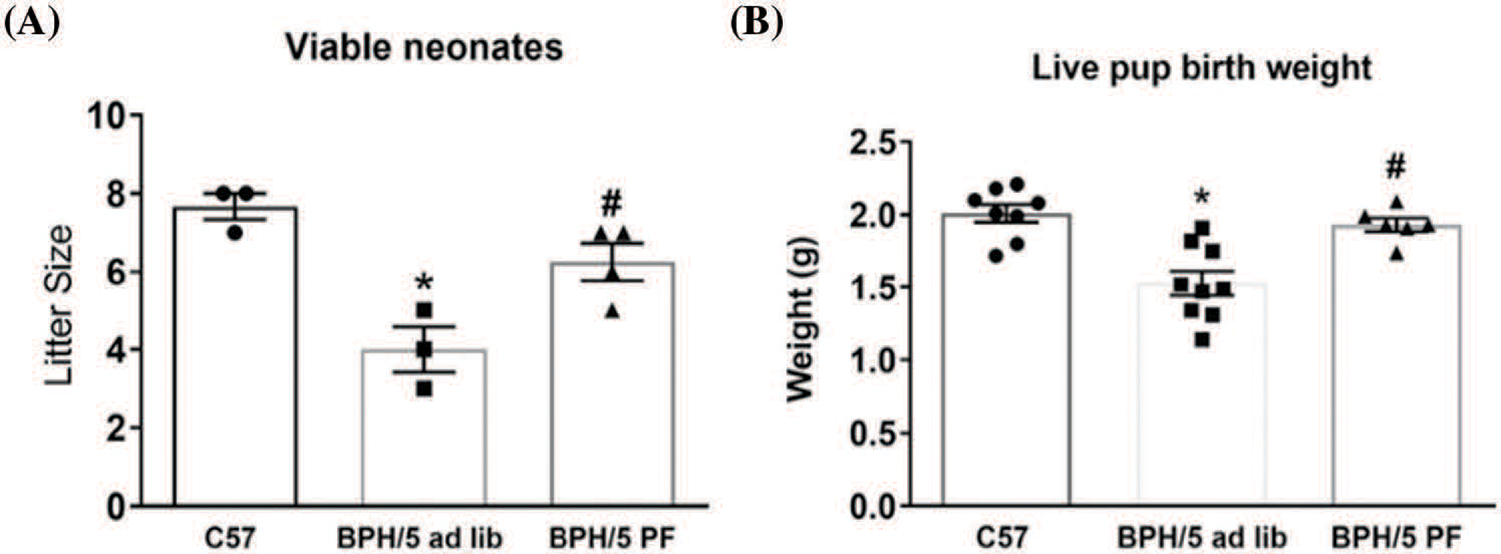
Pair-feeding (PF) BPH/5 throughout pregnancy attenuates fetal demise and growth restriction. (A) Litter size and (B) live pup birth weight (n = 6–9) measured from ad libitum (lib) fed C57 and BPH/5, and BPH/5 PF dams (n = 3–4 dams) *p < 0.05 *vs*. C57 ad lib; ^#^*p* < 0.05 *vs*. BPH/5 ad lib.

At late gestation, e18.5, BPH/5 have significant expansion of the visceral white adipose tissue (WAT) depot around the reproductive tract, which is reduced with pair-feeding in pregnancy by 50% (Fig. 3A). Although pair-feeding BPH/5 only modestly reduced maternal body weight (ad lib: 33.9 ± 1.06 g *vs*. pair-fed: 31.42 ± 1.8 g). Concomitant with reproductive WAT mass in BPH/5 late gestation, placenta lysates have elevated leptin expression compared to C57 as measured by ELISA that is reduced with pair-feeding (Fig. 3B). Previous studies have shown inadequate spiral artery formation and smaller placental vessel area in BPH/5 placenta compared to C57 mice (Dokras et al., 2006). Furthermore, maternal weight loss and reduced leptin have been associated with prevention of FGR in this model (Beckers et al., 2023). To better understand the impact of hypoxia and pair-feeding on fetoplacental development, the placental vessel area in ad lib and pair-fed BPH/5 e18.5 placenta were assessed. Placenta from pair-fed BPH/5 have 2 to 3-fold increase in area of the placental vasculature compared to ab lib BPH/5 mice (Figs. 3C–3E). To further support the impact of BPH/5 adipose tissue reduction and placental leptin in pregnancy with pair-feeding, litter size and pup weight was measured after delivery, and both were significantly higher (Figs. 4A and 4B).

## Discussion

Obesity is thought to contribute to PE. It is hypothesized that an obesogenic maternal environment leads to inadequate trophoblast invasion of the maternal uterine arteries. Impaired placentation with diminished placental vasculature would reduce delivery of oxygen and nutrients to the fetus and promote FGR in PE pregnancies. The role of maternal obesity and specifically leptin in early gestation in PE pregnancies has not been fully described. To better understand this, we utilized pre-hypertensive and obese BPH/5 mice that spontaneously develop a PE-like syndrome. The aim of this study was to provide evidence of pathologic hypoxia and leptin expression in early gestation that precedes impaired fetoplacental development associated with PE in this model. We hypothesized that *ad libitum* fed BPH/5 mice would have elevated hypoxia markers and leptin in implantation sites at e7.5 that is attenuated with maternal weight loss and reduced leptin via pair-feeding.

In this study, the tested markers all respond to hypoxia through different pathways. In the HIF pathway during normoxic conditions, HIFα subunit binds to von-Hippel-Lindau protein, allowing for it to become ubiquitinated and then degraded (Soares et al., 2017). During hypoxic conditions, the HIFα subunit instead stabilizes and interacts with the HIFß subunit and binds coactivators to regulate genes encoding proteins that control adaptions to hypoxia, including VEGF (Soares et al., 2017). Expression of SCF can also be induced by hypoxia and will bind to its receptor, c-kit, to aid in the regulation of placental hemopoietic cell clusters (Broudy, 1997; Sasaki et al., 2010). Ho-1 is a membrane protein that cleaves b-type heme molecules to produce biliverdin, carbon monoxide, and iron as the first rate-limiting step in heme catabolism (Lee et al., 1997). The expression of Ho-1 is induced by stressful conditions, including hypoxia, and the consequent production bilirubin (converted from biliverdin), allows for potent scavenging of free radicals (Foresti et al., 2001). Our results demonstrated a pathologic overexpression of hypoxia markers in *ad libitum* fed BPH/5 e7.5 implantation sites in comparison to physiological expression levels observed in control C57 mice. These markers were reduced with pair-feeding BPH/5 mice. Hypoxia in the decidua in early pregnancy may contribute to *VEGF* overexpression in *ad libitum* BPH/5 e7.5 implantation sites, which similarly normalizes with pair feeding (Olson et al., 2020).

Additionally, we investigated leptin expression during early BPH/5 gestation. Previous studies demonstrated elevated circulating leptin levels in pregnant BPH/5 mice serum (Reijnders et al., 2019), which could be due to adipose tissue and/or placental production of leptin. Therefore, we measured leptin and leptin receptor mRNA expression in e7.5 implantation sites and placental leptin at e18.5. *Ad libitum* BPH/5 e7.5 implantation sites had a greater than 3-fold expression of *Lep* compared to C57 controls, which normalized with pair-feeding. Contrary to *Lep*, *LepR* expression was not significantly elevated in *ad libitum* fed BPH/5 e7.5 implantation sites compared to control mice but did decrease with pair-feeding. These results could be due to the influence of systemic leptin exposure and priming of the maternal decidua early in BPH/5 pregnancy. This is an area of ongoing investigation in the laboratory.

After demonstrating both leptin and hypoxia marker expression was higher in *ad libitum* BPH/5 e7.5 implantation sites compared to *ad libitum* C57 e7.5, we investigated if there was a causal relationship between leptin exposure and hypoxia marker expression in the decidua. Cultured e7.5 decidua explants from only BPH/5 exhibited a statistically significant elevation in hypoxia markers, Hif1a and Ho-1, when cultured with leptin compared to decidua cultures without leptin. The discrepancy whereby BPH/5 but not C57 responded to exogenous leptin *in vitro* may be due to higher leptin sensitivity in BPH/5 tissues from exposure *in vivo*. Ho-1 has been shown to have potential protective capabilities in cells through possible antioxidative, anti-inflammatory, anti-apoptotic effects (Zeng et al., 2013). We speculate that the higher expression of Hif1a with leptin addition could potentiate the expression of Ho-1, which is a downstream target of Hif1a, and have a beneficial effect in BPH/5 mice due to a greater need for cytoprotective properties. It is possible that high levels of Ho-1 is a physiological response that promotes maintenance of pregnancy as BPH/5 mice do have term deliveries. Further investigation is needed to better understand the extent of the effect leptin has on hypoxia marker expression and the impact of this on pregnancy. We briefly tested if transient exposure to hypoxia would induce higher expression of leptin in C57 and BPH/5 decidua compared to normoxic controls. We did not show an elevation of leptin expression in hypoxic decidua suggesting leptin contributes more to expression of hypoxia markers, but these results warrant further experimentation. One hypothesized explanation for the detrimental impact of abnormally upregulated leptin on adverse pregnancy outcomes and PE development is inducible nitric oxide synthase (iNOS) expression. While iNOS is normally expressed in the first half of pregnancy to aid vasodilation and placental development, previous studies on BPH/5 mice have demonstrated an upregulation of iNOS expression (Heyward et al., 2017). Upregulated iNOS has been linked to poor pregnancy outcomes, such as embryo loss. While the intermediate steps are not completely understood, previous rodent studies demonstrated an elevation in leptin leads to increased NO synthesis through elevated iNOS expression (Yang and Barouch, 2007). Other research demonstrated iNOS to be a downstream target of STAT3, which is activated by the Ob-Rb/JAK2 complex that forms when leptin binds to its receptor, though this was seen in astrocytes during transformation (Osuka and Van Meir, 2017). This pathway of activation, from leptin, leptin receptor, Ob-Rb/JAK2, STAT3, leading to iNOS, may explain and contribute to adverse pregnancy outcome seen in the PE-like BPH/5 mice.

To investigate further downstream impacts hypoxia and leptin can have on placental angiogenesis, placental vasculature area was measured. While previous studies have demonstrated *ad libitum* BPH/5 mice exhibit inadequate spiral artery formation and smaller placental vessel area with oxidative stress, in this study pair-fed BPH/5 e18.5 placenta have a greater luminal area of placental vessels in comparison to *ad libitum* fed BPH/5 e18.5 placenta. These results could be associated with aberrant hypoxia and leptin levels in early BPH/5 gestation. Further investigation is warranted to better understand the effect of hypoxia and leptin on the development of PE.

Limitations of this study include the method of weight loss. Restricting the diet of the mice has the potential to place them in a negative energy balance which can allow for the development of adverse fetal outcomes including decreased nephron numbers, hypertension, proteinuria, or renal dysfunction after birth (Luyckx, 2020). While excessive dietary restriction can be harmful, the method of food restriction utilized, pair feeding, which is matched to lean, gestation-matched control mice, acts to normalize obese BPH/5 mice weight and adiposity, and serum leptin by e7.5 (Reijnders et al., 2019), and has demonstrated improvement in fetal and maternal outcomes (Beckers et al., 2023). Pair-feeding BPH/5 showed a similar reduction in serum leptin by e7.5 (83%) as by e18.5 (95%). Although the placenta contributes to circulating leptin more by late gestation than in early, the reduction at e18.5 in BPH/5 may be due to decreased adipose tissue as well as placental expression. This warrants additional exploration to determine the contribution of each organ. Further investigation into hypoxia at the maternal-fetal interface and its effects in the development of PE is necessary. For example, looking into the expression of other markers of hypoxia or proteins in hypoxia-induced pathways, such as hypoxia inducible factor 2a, insulin-like growth factor-II, E-cadherin, or Lysine Demethylase 3A. Another potential area of study includes further localization of the hypoxia at the fetal-maternal interface to determine if it is localized in the fetus, placenta, and/or endometrium. There is also a need to better understand the role of leptin in PE and the exact mechanism behind its elevated expression and its influence at the maternal-fetal interface. Other murine preeclamptic models could also be used to further verify and expand our current findings; such models to consider in future studies include Hif1a knock-out mice or the reduced uterine perfusion pressure (RUPP) model with similar placental ischemia. In conclusion, maternal obesity may contribute to pathologic hypoxia and leptin expression at the fetal-maternal interface during gestation. A reduction in maternal weight and adiposity through pair-feeding normalizes these abnormalities in a mouse model of PE, BPH/5, and the associated angiogenic imbalance. Further studies are required to better understand the impact of abnormal leptin and hypoxia on placental ischemia in PE.

## Figures and Tables

**TABLE 1:** RNA primers used for qRT-PCR

Gene	Forward primer (5′ -> 3′)	Reverse primer (5′ -> 3′)

*18s*	CCGGGCTTCTATTTTGTTGGT	TAGCGGCGCAATACGAATG
*Hif1a*	CCTTAACCTGTCTGCCACTTT	TGCTGCAATAATGTTCCAATTCC
*Ho-1*	GAGAATGCTGAGTTCATG	ATGTTGAGCAGGAAGGC
*Scf*	ATAGTGGATGACCTCGTGTTA	GAATCTTTCTCGGGACCTAAT
*Lep*	TGCTGCAGATAGCCAATGAC	GAGTAGAGTGAGGCTTCCAGGA

## Data Availability

All data generated or analyzed during this study are included in this published article (and its supplementary information files).
